# Cytokine Profile and Oxidative Patterns in Murine Models of Disseminated Infection by Mucorales Species

**DOI:** 10.3390/pathogens14101036

**Published:** 2025-10-13

**Authors:** Hiram Villanueva-Lozano, Martín García-Juárez, Adrián G. Rosas-Taraco, Rogelio de J. Treviño-Rangel, Gloria M. González

**Affiliations:** 1 Servicio de Infectología, Departamento de Medicina Interna, Hospital Regional ISSSTE Monterrey, Monterrey 64380, Mexico; dr.villanueval@hotmail.com; 2 Departamento de Microbiología, Facultad de Medicina, Universidad Autónoma de Nuevo León, Monterrey 64460, Mexico; martin.garciajr@uanl.edu.mx; 3 Departamento y Servicio de Inmunología, Facultad de Medicina, Universidad Autónoma de Nuevo León, Monterrey 64460, Mexico; adrian.rosastr@uanl.edu.mx

**Keywords:** Mucorales, cytokines, reactive oxygen species, Th1 response, Th17 response

## Abstract

Mucormycosis is a life-threatening infection caused by fungi of the Mucorales order, typically associated with immunocompromised hosts, but increasingly reported in immunocompetent individuals. This study investigated fungal burden, Th1/Th17 inflammatory profiles, and organ-specific dynamics in immunocompetent BALB/c mice intravenously infected with *Rhizopus oryzae*, *Mucor circinelloides*, or *Rhizomucor pusillus*. Colony-forming units were quantified in spleen, liver, and kidney at multiple time points, while serum cytokines and oxidative stress markers were analyzed. The results showed fungal persistence primarily in the spleen, accompanied by species-specific Th1/Th17 responses: *R. oryzae* induced the highest inflammatory response among all groups, with maximal cytokine production observed on day 7, particularly for IL-17A (352.58 pg/mL). In contrast, *M. circinelloides* exhibited its peak cytokine levels earlier, reaching the highest TNF-α concentration on day 3 (425.43 pg/mL). Meanwhile, *R. pusillus* triggered an early but moderate inflammatory response, with a maximum TNF-α value of 372.62 pg/mL detected on day 1, followed by clearance. Correlation analysis highlighted distinct immunological patterns, with IL-10 acting as a negative regulator of inflammation, while TNF-α and IL-17A reflected infection intensity depending on species and timing. The spleen emerged as a key organ coordinating immune responses during systemic infection. These findings reveal that mucormycosis in immunocompetent hosts triggers complex, species-dependent immune dynamics beyond classical immunosuppression, emphasizing the need to consider host–pathogen interactions when developing targeted antifungal strategies.

## 1. Introduction

Mucormycosis is a fungal infection caused by various genera of filamentous fungi in the phylum Mucoromycota (formerly Zygomicota), class Mucoromycetes and order Mucorales. These mycoses are classified by the WHO as high priority due to their high resistance to antifungal treatments and their high mortality rate (40–80%) in individuals with comorbidities affecting immune status, such as diabetes mellitus (67%), haematological cancers (22%), and solid cancers (22%). These fungi cause brain and rhino-orbital infections in 56% of cases, sinopulmonary mucormycosis in 31%, and cutaneous, gastrointestinal, and disseminated infections in a lower percentage (13%) [1,2,3]. Among the species most frequently isolated from patients are *Rhizopus oryzae* in 70% of cases [4] followed by *Mucor circinelloides* and *Rhizomucor pusillus* [5].

*R. oryzae* is a globally distributed filamentous saprophyte that exhibits sporangiophores of variable morphology, along with abundant rhizoids and globose, spiny-walled, black sporangia measuring 60 to 180 μm in diameter [6]. Following inhalation, the sporangiospores adhere to the endothelium and branch out, invading blood vessels and leading to thrombosis and vascular tissue degradation [4].

*M. circinelloides* is a fungus capable of sexual and asexual reproduction. Its sporangiophores are sympodially branched and often circinate with spherical sporangia that are variable in size (25–80 μm). A yeast-to-hypha transition occurs under high CO_2_ conditions; nonetheless, it is not considered a dimorphic fungus in its traditional definition. The activation of PKA by cerulenin, cycloleucine, or cAMP is involved in its morphogenesis [7,8]. Though rare, *M. circinelloides* infections resemble *R. oryzae* pathogenesis, typically causing skin or gastrointestinal lesions [9,10]. Its cell wall composition appears central to its unclear virulence mechanisms [11]. 

*R. pusillus* accounts for fewer than 5% of mucormycosis cases [12]. Morphologically, it exhibits characteristics intermediate between *Rhizopus* and *Mucor* species. The sporangiophores are irregularly branched, often arising from stolons located between sporangia with a subglobose to spherical collumela and globose smooth 40–60 µm in diameter sporangium [8,13]. *R. pusillus* displays sensitivity to both osmotic and oxidative stresses [14]. Taxonomically, *R. pusillus* belongs in the order Mucorales but has been relocated from the family Mucoraceae to Lichteimiaceae, distinguishing it from *Rhizopus* and *Mucor* species. Pathologically, it induces necrosis and compromises the integrity of perineural barriers, affecting the pleura, lungs, and nervous tissue [15,16].

The immune system plays a key role in controlling fungal infections, with Th1 and Th2 responses being essential for the containment and resolution of systemic mycoses such as candidiasis and aspergillosis [17,18]. In mucormycosis infection the spores are able to survive and germinate within macrophages, triggering the expression of the transcription factors atf1 and atf2, which regulate germination under acidic conditions. Additionally, activation of the calcineurin signaling pathway contributes to the inhibition of phagosome maturation [19]. Furthermore, a decrease in cytokines associated with Th1 and Th17 responses has been linked to increased susceptibility to cutaneous mucormycosis caused by *R. irregularis* [20,21], underscoring the importance of these pro-inflammatory pathways in host defense against Mucorales. In parallel, enhanced activity of antioxidant enzymes such as superoxide dismutase (SOD) and glutathione peroxidase (GPx) appears to be a key fungal strategy to counteract host defenses mediated by reactive oxygen species (ROS) and oxidative stress [22].

Most mucormycosis immunity research has focused on systemic mycoses or *Rhizopus* spp., leaving responses to other Mucorales understudied. The objective of this study was to evaluate the fungal burden in the liver, spleen, and kidneys [8]; analyze Th1/Th17 cytokine profiles; and assess oxidative stress via SOD and GPx activity in immunocompetent mice infected with *R. oryzae*, *M. circinelloides*, or *R. pusillus*. The null hypothesis is finding no measurable relevant differences in these variables. These insights will help define baseline immunity and identify potential therapeutic targets.

## 2. Materials and Methods

### 2.1. Fungal Strains

The mucoralean fungi used in this work were isolated and collected in the Departamento de Microbiología, Universidad Autónoma de Nuevo León in Mexico. The strains were identified based on microscopic morphology and confirmed by ITS and D1/D2 sequencing: *Rhizopus oryzae* (GenBank codes: MK174988 and MK796452), *Mucor circinelloides* (GenBank codes: MK174983 and MK174982) and *Rhizomucor pusillus* (ATCC 46342 NR_111462).

### 2.2. Animals

Male BALB/c mice (6 weeks old, weight, 22–25 g; Harlan, Mexico City, Mexico) were used for all experiments. Animals were kept in groups of five in ventilated cages at the animal facility. Purified water and chow diet were provided ad libitum. Experiments complied with Mexican animal regulations (NOM-062-ZOO-1999) and were approved by the Ethics Committee of the School of Medicine of the Universidad Autonoma de Nuevo Leon. (MB18-00006), conducted in a certified facility (SAGARPA-SENASICA AUT-B-B-0425-114).

### 2.3. Mouse Mucormycosis Models

Inocula were freshly prepared on the day of each infection. Five-day cultures on potato dextrose agar (PDA) were flooded with sterile saline supplemented with 0.1% Tween 80. Sporangiospores were then harvested by scraping the agar surface to detach the fungal cells, washed twice and filtered through sterile gauze to remove clumps of sporangiospores, hyphae and agar particles. The resulting suspensions were adjusted to 1.5 × 10^4^ sporangiospores/mL for each experimental model, based on hemocytometer counts, and were serially inoculated onto PDA to confirm viability.

For each experimental infection, a total of 22 mice per strain were individually restrained into a rotating tail injector (Braintree Scientific Inc., Braintree, MA, USA) and were challenged with 0.2 mL of the adjusted inoculum, injected via the lateral tail vein based on other murine models of disseminated mucormycosis infection [23]. Four uninfected animals were included as controls for each infection. Before the inoculation, groups of six mice were randomly selected and euthanized by cervical dislocation after supervised inhalation of 5% isoflurane (Fluriso, Boise, ID, USA). The sample size was established considering financial constraints and the requirements set by the ethics committee. The mice were euthanized on days 1, 3 and 7 post-infection. Animals were monitored at least twice daily for the development of clinical symptoms and euthanized if they met predefined endpoints (≥25% weight loss, prolonged lethargy, severe dyspnea, inability to move coordinately and lack of response to tactile stimuli) or at the end of the experiment.

### 2.4. Fungal Tissue Burden

Spleens, kidneys and livers of euthanized mice were aseptically collected during animal necropsy, weighed and placed within Whirl-Pak plastic sample bags (Fisher Scientific^®^, Waltham, MA, USA). Organ tissues were gently homogenized in 2 mL of sterile saline supplemented with chloramphenicol (Pisa^®^, Mexico City, Mexico) and gentamicin (Pisa^®^, Mexico City, Mexico). Aliquots were taken and serially diluted for quantitative culture in PDA. Plates were incubated at 30 °C for up to 72 h. Fungal colonies were enumerated and CFU per gram of tissue was calculated.

### 2.5. Cytokine Profiling

After anesthesia through isoflurane inhalation, the blood of each mouse was extracted directly from the murine heart by cardiac puncture. Samples were collected in Microtainer tubes (Becton Dickinson^®^, Franklin Lakes, NJ, USA) and centrifuged at 1700× *g* for 10 min at 4 °C. Serum was separated and samples were immediately aliquoted and preserved at −80 °C until use.

To compare the murine inflammatory responses induced by the three fungal strains, the protein levels of interleukin-1β (IL-1β), interleukin-6 (IL-6), interleukin-10 (IL-10), interleukin-17A (IL-17A), interferon-γ (IFN-γ), and tumor necrosis factor-α (TNF-α) were quantified in serum by the Bio-Plex Pro Mouse Cytokine Th17 6-Plex Panel A (Bio-Rad^®^, M6000007NY) using the Bio-Plex 200 System (Bio-Rad^®^, Hercules, CA, USA), according to the manufacturer’s instructions.

### 2.6. Oxidative Stress Biomarkers

Samples of spleen, kidney and liver homogenates previously collected were twice washed with sterile saline solution to reduce the erythrocytes present in the sample. The material was kept in an ultra-freezer at −80 °C until use. At the time of use, a new wash with sterile saline solution was performed. A 10 μL aliquot of each sample was used to measure GPx activity using the commercial RANSEL kit (Randox^®^ laboratories, Crumlin, UK), the indications for realization as described by the supplier were followed, and the activity of GPx was spectrophotometrically measured at 340 nm. For SOD, 10 μL of each sample was processed using the commercial RANSOD kit (Randox^®^ Laboratories, Crumlin, UK). SOD activity was measured in a spectrophotometer at 505 nm.

### 2.7. Statistics

The results are expressed as the mean ± standard error of the mean (SEM). Statistical analyses and figure generation were conducted using GraphPad Prism^®^ version 8.0 (GraphPad Software, La Jolla, CA, USA). Data normality was assessed using the Shapiro–Wilk test. For datasets with a normal distribution, a one-way analysis of variance (ANOVA) followed by Dunnett’s multiple comparisons test was applied. In the case of non-normally distributed data, the Kruskal–Wallis test was performed, followed by Dunn’s multiple comparisons test. Oxidative stress parameters were analyzed using two-way ANOVA with Tukey’s post hoc test. Statistical significance is indicated by asterisks: * *p* < 0.05; ** *p* < 0.01; *** *p* < 0.001; and **** *p* < 0.0001. Pearson correlation analysis assessed linear relationships between continuous variables, calculating correlation coefficients (r) and *p*-values. Positive (blue) and negative (red) correlations were found between fungal burden and cytokine levels. An r of +1 indicates a perfect positive correlation, −1 a perfect negative correlation, and 0 no correlation.

## 3. Results

### 3.1. Organ-Specific Fungal Loading During Systemic Mucormycosis in Immunocompetent Models Reveals Splenic Predominance

**Figure 1:**
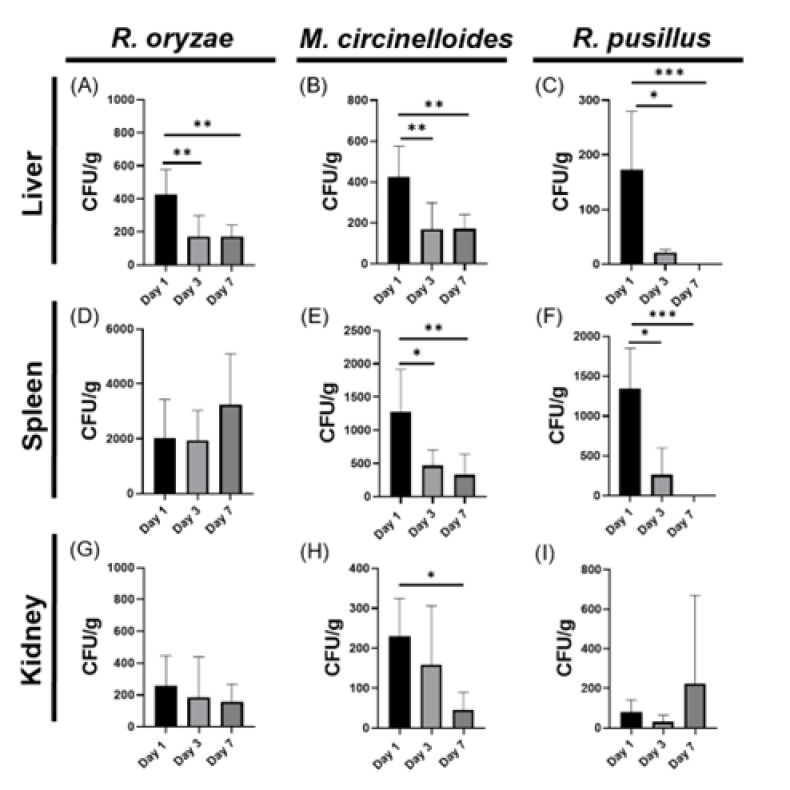
Distinct fungal dissemination dynamics across organs and time points for each mucoralean species (*Rhizopus oryzae*, *Mucor circinelloides*, and *Rhizomucor pusillus*). Bar graphs illustrate the CFU load for liver (**A**–**C**), spleen (**D**–**F**), and kidney (**G**–**I**), with values shown as mean ± SEM. Statistical significance was assessed using one-way ANOVA followed by Dunnett’s multiple comparisons test: *p* < 0.05 (*), *p* < 0.01 (**), *p* < 0.001 (***).

To assess the effect of systemic infection by Mucoralean fungi in immunocompetent mice, we measured fungal loading by quantifying colony-forming units (CFU) in liver, spleen and kidney. In mice infected with *R. oryzae*, a significant increase in the fungal load in the liver was observed on day 1, which decreased on day 3 (Figure 1A); in the spleen, only an increasing trend of CFU was observed on day 7 (Figure 1D), while no differences in kidney were observed (Figure 1G). In *M. circinelloides*-induced infection, a similar pattern was observed in the liver, spleen and kidney (Figure 1B,E,H), where there was a significant increase in fungal load on day 1 and then decreases on days 3 and 7. Meanwhile, in mice infected with *R. pusillus*, we detected a significant increase in fungal load on day 1 in liver and spleen, which decreased on day 3 and was not detectable on day 7 (Figure 1C,F). Additionally, in the kidney we observed an increase in CFU on day 7, but it was not significant. It should be noted that the fungal load was consistently higher in the spleen than in the liver and kidney of mice infected with all three Mucorales species (Figure 1D–F). These findings suggest that the spleen plays an important role in colony growth during infections of immunocompetent mice.

### 3.2. Specific Dynamics of the Immune Response to Mucoralean Infections with Variations According to Fungus and Postinfection Time

**Figure 2:**
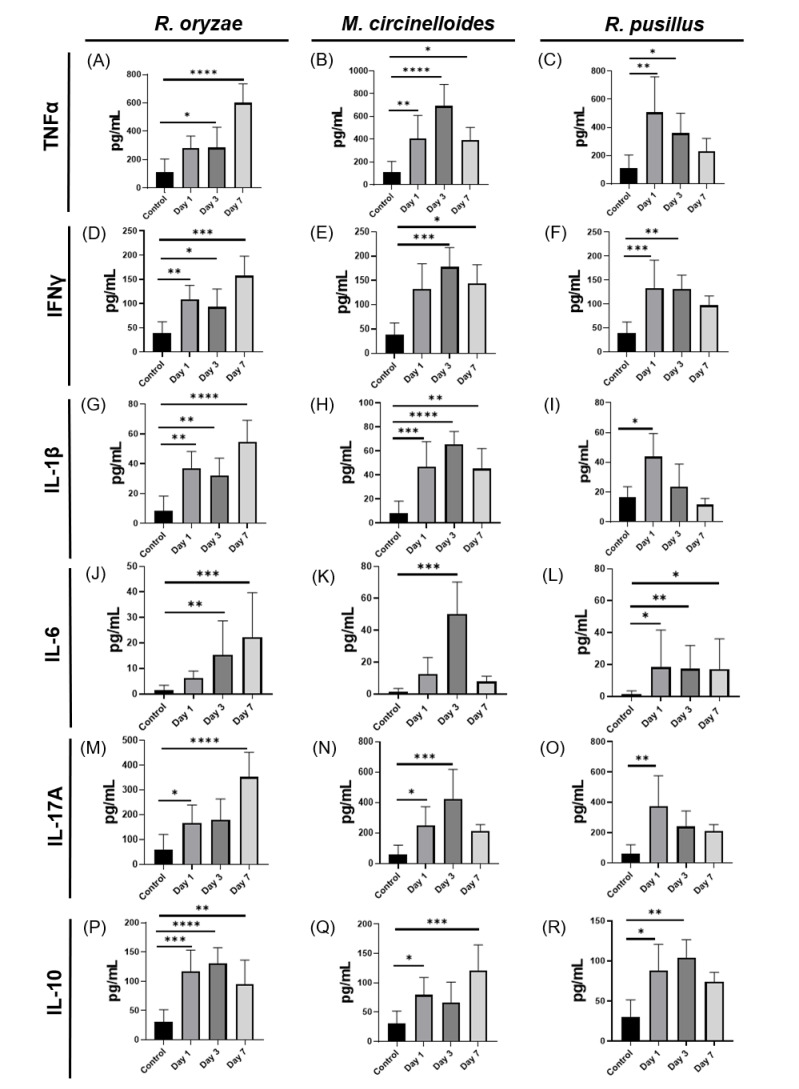
Time-dependent immune response induced by mucoralean infection is predominantly mediated by Th1 and Th17 cytokines. The bar graph displays serum cytokine levels (pg/mL) at three different time points during mucoralean infection. For *R. oryzae*, Th1 (**A**,**D**), Th17 (**G**,**J**,**M**), and IL-10 (**P**) were analyzed. For *M. circinelloides*, Th1 (**B**,**E**), Th17 (**H**,**K**,**N**), and IL-10 (**Q**) were measured. For *R. pusillus*, Th1 (**C**,**F**), Th17 (**I**,**L**,**O**), and IL-10 (**R**) were quantified. Statistical significance was determined by one-way ANOVA followed by Dunnett’s multiple comparisons test: *p* < 0.05 (*), *p* < 0.01 (**), *p* < 0.001 (***), *p* < 0.0001 (****).

Once the fungal load on the organs was evaluated, the effect of infection with *R. oryzae*, *M. circinelloides* and *R. pusillus* on the expression of cytokines of the Th1 profile (TNF-α and IFN-γ), cytokines of the Th17 profile (IL-6, IL-1β and IL-17A), and the levels of IL-10, a cytokine that plays an important role in the modulation of the inflammatory response, were measured. Mice infected with *R. oryzae* showed a gradual increase in cytokines of the Th1 and Th17 profile from day 1 to day 7, showing a highly significant increase compared to controls and the others on the seventh day after infection (Figure 2A,D,G,J,M). On the other hand, we observed an increase in IL-10 with a pattern opposite to that of the pro-inflammatory cytokines (Figure 2P). In mice infected with *M. circinelloides*, an increase in pro-inflammatory cytokines was observed mainly on day 3 post-infection. An increase was found in TNF-α and IFN-γ (Figure 2B,E) as well as in IL-6, IL-1β and IL-17A (Figure 2H,K,N). On the other hand, IL-10 showed an increase only on days 1 and 7 (Figure 2Q). These findings suggest that the peak pro-inflammatory response in *M. circinelloides* infection occurs on day 3, the day with the lowest level of IL-10 (Figure 2Q). Finally, *R. pusillus* showed an acute pro-inflammatory response, as cytokines of the Th1 profile (Figure 2C,F) and Th17 profile (Figure 2I,O,R) increased on day 1. In contrast, IL-10 increased on day 3 and decreased after day 7 (Figure 2R). Therefore, the previously observed decrease in fungal load (Figure 1C,F) on day 7 suggests that the immune response to *R. pusillus* is more prominent on day 1 and subsequently decreases on day 7, allowing these organisms to undergo a possible resolution process.

### 3.3. Correlations Between Fungal Load and Cytokines Reveal Organ- and Fungus-Specific Immune Patterns During Mucormycosis in Immunocompetent Mice

**Figure 3:**
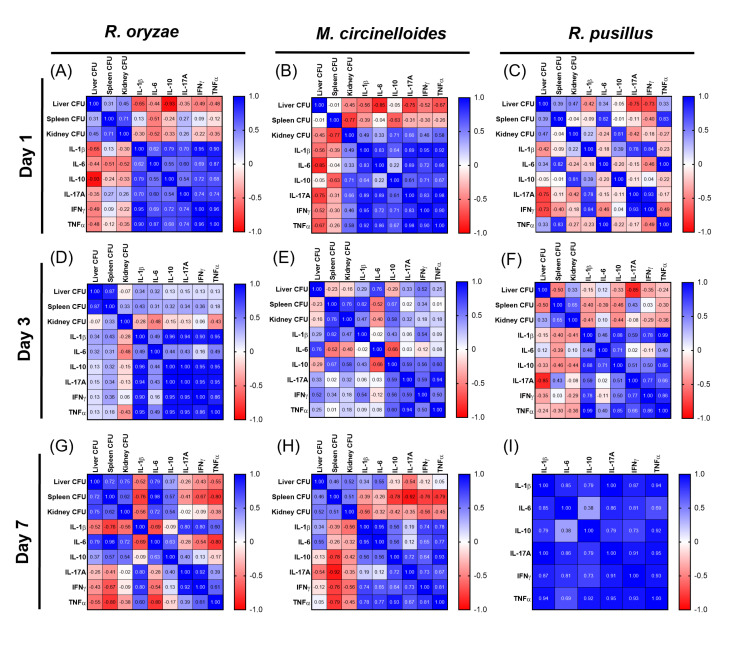
Correlation between fungal burden in different organs and levels of pro-inflammatory and anti-inflammatory cytokines during murine infection by Mucorales. The scatter plot illustrates the correlation between fungal burden in various organs and cytokine levels at three time points during infection. Panels (**A**,**D**,**G**) correspond to *Rhizopus oryzae*, panels (**B**,**E**,**H**) to *Mucor circinelloides*, and panels (**C**,**F**,**I**) to *Rhizomucor pusillus*. To visualize the correlation patterns, heat maps were generated, with positive correlations displayed in blue and negative correlations in red. The Pearson correlation coefficient quantifies both the strength and direction of the relationship: values closer to +1 indicate a strong positive correlation, while values closer to −1 indicate a strong negative correlation. A coefficient of 0 indicates no correlation between the variables.

After measuring the fungal load and evaluating the cytokine profile, we evaluated the correlation between the CFU count in liver, spleen and kidney with plasma cytokine levels on days 1, 3 and 7 post-infection with all three mucoralean species. First, we observed that during *R. oryzae* infection on day 1, the fungal load in the liver showed a strong negative correlation with IL-10 (−0.93), indicating that as the fungal load in the liver increases, the levels of this anti-inflammatory cytokine decrease (Figure 3A). However, a moderate negative correlation was also observed in the levels of proinflammatory cytokines such as IL-1β (−0.65) and TNF-α (−0.48), so there was no clear pattern of immune regulation in this organ. On the other hand, there was a weak positive correlation with IL-17A in spleen (0.27) and kidney (0.26) (Figure 3A). On day 3 of infection by *R. oryzae*, the CFU in liver and spleen exhibited weak positive correlations in all cytokines evaluated, with IL-1β (0.43) in spleen being the most prominent. On the other hand, cytokines in the kidney show a negative trend, such as IL-6 (−0.48), indicating an association between the spleen and a pro-inflammatory response (Figure 3D). The highest levels of Th1 and Th17 cytokines induced by *R. oryzae* were observed on day 7 (Figure 2A,D,G,J,M); however, IL-6 was found to be the most positively correlated in liver (0.79), spleen (0.98) and kidney (0.72), while in the spleen, a strong negative association with TNF-α (−0.80) was found (Figure 3G). These results showed that the fungal load does not exhibit a determining relationship with the previously observed inflammatory profile.

On day 1 of the *M. circinelloides* infection, lower levels of IL-6 (−0.85), IL-17A (−0.75) and lower TNF-α (−0.67) were observed with a higher fungal load in the liver; a similar pattern was also obtained in the spleen, but with a weak correlation. On the other hand, in the kidney, IL-10 (0.71) showed a positive correlation, suggesting that an increase in the fungal burden triggered a regulatory response (Figure 3B). On day 3, *M. circinelloides* infection showed the most pronounced increase in immune response (Figure 2B,E,H,K,N,Q) and the plasma cytokine correlation matrix revealed that an increase in fungal load showed a weak to moderate positive correlation in the three organs, highlighting the strong correlations of IL-6 in liver (0.76) and IL-1β (0.82) in spleen, which corresponds to the immune profile shown above (Figure 3E). Furthermore, on day 7, a higher negative correlation was observed between the three organs and cytokines, showing that an increase in fungal load is significantly associated with decreases of IL-10 (−0.78), IL-17A (−0.92), IFN-γ (−0.76) and TNF-α (−0.79) in the spleen. These results suggest that for *M. circinelloides* infection, the fungal charge in the spleen is important for modulation of the immune response.

In mice infected with *R. pusillus*, we observed that on day 1 there was a strong negative correlation between fungal load and IL-17A (−0.75) and IFN-γ (−0.73) in the liver (Figure 3C). In the spleen, the increase in CFU increased with levels of IL-6 (0.82) and TNF-α (0.83), which shows that the increase in CFU in the spleen is associated with the immune profile previously observed during infection by this fungus. Whereas in kidney at day 1, IL-10 (0.81) was found to have a highly significant correlation (Figure 3C). By day 3, the increase in fungal load in liver had a strong negative correlation with the proinflammatory cytokine IL-17A (−0.85), which suggests the resolution of this infection (Figure 3F), since on day 7 no fungal load was detected in the infection by *R. pusillus* (Figure 1C,F).

The correlations between fungal and cytokine load varied according to the fungus and organ. *R. oryzae* showed inconsistent immune responses, *M. circinelloides* involved the spleen in immune modulation, and *R. pusillus* presented an initial inflammatory response associated with the spleen; there was a variation in the immune response across organs and a progressive decline in fungal burden that became undetectable by day 7, suggesting effective infection control. These results highlight the role of fungal loading in the spleen associated with inflammatory regulation in immunocompetent mice.

### 3.4. Differential Regulation of Inflammatory Cytokines and Inflammation Patterns Modulated by Fungal Burden and Infection Time Across Three Mucoralean Species

**Figure 4:**
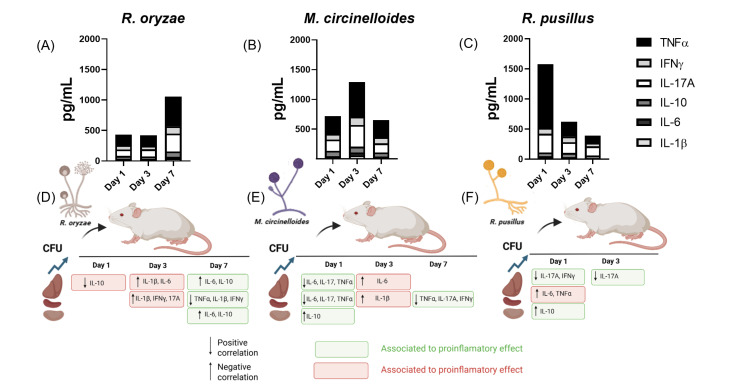
Temporal dynamics of pro-inflammatory and anti-inflammatory responses during Mucoralean infection and their correlation with fungal burden. Bar graphs depict immune profiles during Mucorales infections, with cytokine levels on the *Y*-axis and time points (days 1, 3, and 7 post-infection) on the *X*-axis. Panels (**A**–**C**) show the profiles for *R. oryzae*, *M. circinelloides*, and *R. pusillus*, respectively. The color of the boxes represents the association between fungal burden and the levels of Th1, Th17, and IL-10 cytokines with the inflammatory response in plasma. Red boxes indicate pro-inflammatory effects, while green boxes represent anti-inflammatory effects. Panels (**D**–**F**) display the corresponding correlation patterns. Arrows indicate the direction and strength of the correlations between fungal load and plasma cytokine levels.

To compare the effects induced by different mucoralean infections on cytokine pro-files at days 1, 3, and 7 post-infections in immunocompetent hosts, and to integrate the correlation analyses previously described between cytokine levels, time points, and target organs, we present the data shown in Figure 4A–C. These figures show that all three species induce an increase in pro-inflammatory cytokines, particularly TNF-α and IL-17A. Notably, each infection exhibited a distinct temporal pattern of inflammation: *R. oryzae* showed a pronounced pro-inflammatory response on day 7 (Figure 4A), *M. circinelloides* on day 3 (Figure 4B), and *R. pusillus* on day 1 (Figure 4C). In parallel, we integrated the cytokine correlation patterns derived from Pearson analysis, classifying cytokines by their systemic pro-inflammatory (red) or anti-inflammatory (green) effects in order to visually represent organ- and time-specific relationships across the three infections.

During *R. oryzae* infection, a negative correlation (downward arrow) was observed on day 1 between fungal burden in the liver and IL-10 levels in the same organ. By day 3, higher fungal loads correlated with increased IL-6 and IL-1β in both the liver and spleen, suggesting a systemic pro-inflammatory response associated with disease progression (Figure 4D). Interestingly, by day 7, the fungal burden showed negative correlations with pro-inflammatory cytokines in the liver and spleen (Figure 4A), while a positive correlation was observed with plasma anti-inflammatory cytokines across all three organs, indicating that at this later stage, the fungal burden is not associated with the systemic response (Figure 4). In mice infected with *M. circinelloides*, negative correlations for IL-6, IL-17A, and TNF-α were observed in the liver and spleen on day 1, alongside a positive correlation between IL-10 and fungal load in the kidney (upward arrow), suggesting an early systemic anti-inflammatory effect (green). However, by day 3, an increased fungal burden in the liver and spleen was associated with a pro-inflammatory cytokine profile, consistent with plasma cytokine patterns (Figure 4B). On day 7, a negative association between splenic fungal burden and systemic Th1 and Th17 cytokines was observed, indicating a reduction in systemic inflammation (Figure 4E). In the case of *R. pusillus* infection, where the increase in Th1 and Th17 plasma cytokines is pronounced, it occurred as early as day 1 and the fungal burden in the spleen was positively correlated with this early inflammatory response. Whereas liver and kidney showed inverse associations (Figure 4F).

These patterns suggest that, in the context of these infections, the fungal burden in target organs correlates specifically with the peaks of Th1 and Th17 cytokine responses: in both liver and spleen on day 3 in *M. circinelloides*, and exclusively in the spleen on day 1 in *R. pusillus*. In contrast, during *R. oryzae* infection, the fungal burden was not associated with the systemic pro-inflammatory response on its day 1 cytokine peak.

### 3.5. Comparative Response to Oxidative Stress Induced by Mucoralean Infection

**Figure 5:**
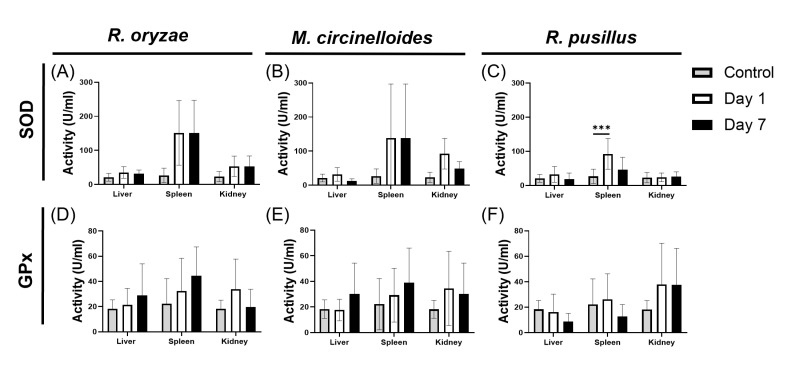
Evaluation of oxidative stress response in murine models infected with Mucorales. Bar graphs display superoxide dismutase (SOD) activity (*Y*-axis) across different organs (*X*-axis) at day 0 (control), day 1, and day 7 post-infection for *R. oryzae* (**A**), *M. circinelloides* (**B**), and *R. pusillus* (**C**). Additionally, the activity levels of glutathione peroxidase (GPx) are shown for *R. oryzae* (**D**), *M. circinelloides* (**E**), and *R. pusillus* (**F**). Oxidative stress parameters were analyzed using two-way ANOVA with Tukey’s post hoc test *p* < 0.001 (***).

To assess how infection by Mucoralean fungi impact oxidative stress, we measured the activity of the antioxidant enzymes GPx and SOD, which help counteract oxidative damage by neutralizing free radicals and peroxides. In infections caused by *R. oryzae* (Figure 5A) and *M. circinelloides* (Figure 5B), SOD activity remained unchanged across all organs examined on days 1 and 7 post-infection (Figure 5C). In contrast, spleens from mice infected with *R. pusillus* showed a significant increase in SOD activity on day 1, which coincided with the early peak in inflammatory cytokines observed in this infection (Figure 2C,F,I,O). Although the kidney was the only organ where the fungal burden persisted on day 7, no corresponding increase in SOD activity was detected. These findings suggest that oxidative stress responses may contribute to the early control or resolution of *R. pusillus* infection, distinguishing it from the other two species. In contrast, GPx activity remained stable, with no significant differences detected in any organ at any time point across all three fungal infections (Figure 5D–F).

## 4. Discussion

Mucormycosis is a severe fungal infection primarily affecting immunocompromised hosts, but increasingly affecting all kind of patients, caused by fungi of the Mucorales order. We investigated the effects of *R. oryzae*, *M. circinelloides*, and *R. pusillus* infections in immunocompetent mice, measuring fungal burden in the liver, spleen, and kidney on days 1, 3, and 7 post-infection, and assessing serum Th1 and Th17 responses. The spleen showed the highest fungal load across all species, accompanied by distinct inflammatory profiles sometimes reaching more than 10 times the fungal load of other organs. Interestingly, a study report using an inoculum of 10^5^ spores/5 mL of the *R. oryzae* E99-880 isolate in diabetic BALB/c mice (induced with streptozotocin at 210 mg/kg) reported an increase in fungal burden between 6- and 24-h post-inoculation, as measured by qPCR and CFU quantification, in the kidney, spleen, liver, and brain. Although fungal load was lower in the liver and spleen, it was higher in the brain and kidney, without further progression over time [24]. In our immunocompetent mouse model, tissue-specific fungal dynamics showed a comparable trend across the three species, particularly the early and sustained decrease in liver fungal burden from day 1 onward. In other study, BALB/c immunocompetent mice infected intravenously and retro-orbitally with 1.25 × 10^6^ sporangiospores/100 μL phosphate buffered saline (PBS) of the *M. circinelloides* 1006PhL strain were reported to exhibit a fungal load exceeding 10^4^ CFUs in the liver, spleen, and kidney. In contrast, our data revealed a lower fungal burden, with approximately 2 × 10^3^ CFUs in the spleen. However, the referenced study does not specify the timing of fungal load assessment relative to the infection [25]. We inoculated 1.5 × 10^4^ sporangiospores/mL of *R. pusillus* and observed that by day 7, no CFUs were detectable in the liver or spleen. This suggests that infections caused by this fungus may yield false-negative culture results due to a fungal burden too low for detection via CFU counts. For instance, a patient with myeloid leukaemia exhibited liver and splenic lesions on a CT scan, along with histological findings (aseptate hyphae) indicative of mucormycosis. Although cultures were negative, *R. pusillus* was identified through pan fungal PCR and sequencing of the ribosomal ITS2 and LSU regions. This underscores the limitations of standard culture techniques in detecting low fungal burdens in certain organs [26]. Additionally, studies involving other Mucorales of the *Lichtheimiaceae* family, such as *Lichtheimia corymbifera*, reported that sporangiospore germination and lesion development were influenced not only by the inoculum concentration (1 × 10^9^ and 1 × 10^11^ in 5 mL of RPMI medium with 10% FBS) but also by the preparation of the medium. Greater effects were observed with supplementation using YPD (yeast peptone dextrose) and RPMI media, likely due to the high energy demands of the fungus [27]. *R. pusillus* may cause an acute, aggressive infection in immunocompromised hosts, with peak effects in immunocompetent hosts likely occurring within the first days, explaining the undetectable tissue burden by day 7 [28].

The immune response to Mucorales has been less studied, as research has predominantly focused on *Candida* spp. and *Aspergillus* spp., where Th1 and Th2 cytokines play central roles in the immune response [29,30,31]. In both fungal infections, the immune response includes a Th1 response mediated by CD4, CD8, and NK cells [32], with TNF-α secretion also by macrophages, neutrophils, and dendritic cells [32]. The Th17 response is characterized by the pro-inflammatory cytokines IL-17A, IL-1β, and IL-6, which can exert either pro- or anti-inflammatory effects depending on the context, such as the type of secreting cell and its receptor (soluble or membrane-bound) [33,34]. In non-lethal systemic *Aspergillus fumigatus* infection models using C57BL/6 mice, a progressive decrease in tissue fungal burden has been correlated with increased mRNA expression of IL-17 and IFN-γ in the spleen on days 3, 7, and 15 post-infection. In contrast, IL-4 expression in splenic cells remained significantly lower throughout the infection, resulting in elevated IFN-γ/IL-4 and IL-17/IL-4 ratios [35].

Pathogenicity of Mucorales relies on their ability to invade blood vessels through the interaction between CotH proteins and the GRP78 receptor. This interaction in endothelial cells is enhanced by hyperglycemia, iron overload, and the production of ketone bodies, which damage the extracellular matrix and facilitate tissue invasion [36,37,38,39] and may also contribute to immune evasion, as Mucoralean fungi have been described as capable of germinating within macrophages and inhibiting phagosome maturation pathways [19]. Regarding the adaptive immune response, it is known that patients with mucormycosis caused by *R. oryzae,* and *R. pusillus* exhibit an immune profile driven by lymphocytes, macrophages, and polymorphonuclear neutrophils (PMNs), triggering responses involving IFN-γ, IL-17A, and IL-10 [40,41]. Additionally, NK cells and T cell-mediated responses to *R. oryzae*, *L. ramosa*, *L. corymbifera*, *M. circinelloides*, and *R. microsporus* play a role. This mechanism involves the recruitment of PMNs to induce hyphal damage, the release of IL-2 and IL-7 to expand specific T cells, and the production of IL-13, IL-5, TNF-α, and IL-10. Furthermore, IL-17A and IL-23 are important for dendritic cell activation [42]. *R. oryzae* has been reported to promote an increase in the expression of IL-1β and TNF-α mRNA at 24 h in PMN and oxidative stress mediated impaired extracellular release of O_2_ [43]. Additionally, an increase in Th1-type cytokines was observed in dendritic cells infected with *R. oryzae* [44]. Our results show that *R. oryzae* triggers a time-dependent production of cytokines Th1 and Th17 mainly on day 7. Previously, in human lymphocyte culture, this fungus was shown to induce a Th1 response that promoted cross-reactivity with *Mucor* species [45]. Other studies support these findings. For instance, research using immunocompetent C57BL/6 and BALB/c mice demonstrated that the resolution of infection occurred on day 16, primarily mediated by the production of IL-17A and IL-2. Moreover, mice lacking IL-17 showed impaired resolution of the infection [46]. Furthermore, we observed an increment of the regulatory cytokine IL-10, an anti-inflammatory cytokine that inhibits IL-1β, TNF-α and IL-6. This discrepancy has been explained due to the culture of lymphocytes infected with *R. oryzae* showing a different profile when stimulated by other cytokines, such as IFN-γ, which promotes the release of IL-10 [47]. For *M. circinelloides*, the information is limited. In a zebrafish model, *Mucor* sporangiospores induce the expression of TNF-α and IL-1β dependent on the viability of the sporangiospores in the posterior brain infection model [48]. The role of pro-inflammatory cytokines in specific immune responses has not been described at the time of this publication; only their effect on phagosome inhibition and cell death induced in macrophages has been reported [19].

The data regarding infection due to *R. pusillus* is also scarce, representing less than 1% of reported cases of mucormycosis [26], and in 2020, only 28 cases were published [49]. It has been communicated that a patient showed an increase in T cells and production of IL-10 and IL-4 [40]. In a study conducted in BALB/c mice, the effect of *R. pusillus* inoculum (2 × 10^6^ sporangiospores/mL) on the production of IL-22, IL-17A and IFN-γ in skin lesions was evaluated. They observed that the main production of IFN-γ occurred between day 2 and 3 after challenge (200 pg/mL), while we obtained in plasma 120 pg/mL mainly between day 1 and 3. On the other hand, they obtained an average of about 300 pg/mL of IL-17A and we obtained 400 pg/mL. However, they note that it mainly stimulates the production of IL-22 [50].

The liver plays a central role in metabolic functions, while the spleen is a key immunological organ. Previous studies have shown that impaired liver function can increase susceptibility to infection by fungi of the Mucorales order [51,52,53], whereas the spleen plays an important role in the immune response to other filamentous fungal infections [35]. Our correlation analyses indicate that Mucoralean infection is associated with a higher fungal burden in the liver and spleen, accompanied by elevated IL-1β levels and a pro-inflammatory response—particularly on days 3 and 7 post-infection with *R. oryzae,* and on day 3 in the liver with *Mucor circinelloides*. In contrast, *R. pusillus* infection was associated with systemic inflammation, primarily involving the spleen. These responses may be linked to the subsequent progressive reduction in fungal burden observed at later time points. Taken together, these findings suggest that the fungal load in the liver and spleen may modulate both direct and indirect components of the immune response. Nonetheless, further studies are required to elucidate the underlying mechanisms.

The production of ROS is a key defense mechanism against fungal infections. Primarily driven by macrophages, ROS inhibits hyphal growth by inducing oxidative stress, which is toxic to both fungal cells and host tissue [54]. Some fungi are known to counteract this oxidative damage by triggering an antioxidant response mediated by enzymes such as SOD, which converts superoxide (O_2_^−^) into hydrogen peroxide (H_2_O_2_), and GPx, which further reduces H_2_O_2_ to water using reduced glutathione as a cofactor, thereby diminishing ROS levels [55]. In other fungal species, such as *Candida* spp., upregulation of SOD represents a primary defense mechanism against free radicals [56]. In our study, SOD and GPx activity measured in the infected organs increased toward day 7 but it did not reached statistical significance, likely due to host cytotoxicity. However, in the spleen on day 1 post-infection with *Rhizomucor pusillus*, intense inflammation coincided with a significant elevation of SOD and with a higher fungal burden, possibly reflecting the fungus’s response to early host oxidative stress.

In line with our findings, numerous reports have addressed the previously mentioned characteristics of these *Mucorales* fungi. Efforts to improve the therapeutic outcome have included immune augmentation strategies, such as the use of interferon-γ and granulocyte–macrophage colony stimulating factor, the change in the local microenvironment through the use of hyperbaric oxygen and even the use of a humanized antibody against mucormycosis targeting angioinvasion [57,58,59].

Some of the limitations of our study are the short evaluation time of the experiment and the fact that some parameters assessed do not represent the multiple components of the immune response to fungi. Nonetheless, the cytokine profiles dynamically presented deepen our understanding of immune responses and the link between fungal burden and inflammation during infection by mucoralean fungi in immunocompetent mice.

## 5. Conclusions

This study showed that systemic mucormycosis in immunocompetent mice triggers species and organ specific immune responses. *R. oryzae* induces a sustained Th1/Th17 response (peak day 7), *M. circinelloides* peaks early (day 3), and *R. pusillus* elicits an acute response (day 1). The spleen, with the highest fungal burden, is probably central to immune regulation to this fungal infection. Key cytokines (TNF-α, IL-17A, IL-1β) and oxidative stress markers vary by species and time. These findings highlight distinct immune dynamics and the value of animal models of fungi infection.

## Data Availability

The data presented in this study are available on request from the corresponding author due to privacy restrictions established by the Microbiology Department.

## Abbreviations

The following abbreviations are used in this manuscript.

SOD	Superoxide dismutase
GPx	Glutathione peroxidase
PBS	Phosphate buffered saline
ITS	Internal transcribed spacer
D1/D2	26S ribosomal DNA region
PDA	Potato dextrose agar
RPMI	Roswell Park Memorial Institute
PKA	Protein kinase A
ROS	Reactive oxygen species
CotH	Coat protein homolog
CFU	Colony-forming units
